# SRSF6 is upregulated in asthmatic horses and involved in the MYH11 SMB expression

**DOI:** 10.14814/phy2.13896

**Published:** 2018-10-22

**Authors:** Mohamed Issouf, Amandine Vargas, Roxane Boivin, Jean‐Pierre Lavoie

**Affiliations:** ^1^ Department of Clinical Sciences Faculty of Veterinary Medicine Université de Montréal Saint‐Hyacinthe Quebec Canada

**Keywords:** Airway smooth muscle, alternative splicing, asthma, hnRNPA1, SMB isoform, SRSF1, SRSF6

## Abstract

Smooth muscle has a central role in bronchospasm‐induced airway obstruction in asthma. Alternative mRNA splicing of the smooth muscle myosin heavy chain (myh11) gene produces four different isoforms, one of which (SMB) is characterized by the inclusion of the exon5b, which doubles the smooth muscle cells contraction velocity. Deciphering the regulation of the expression levels of the SMB isoform would represent a major step for the understanding of the triggers and pathways leading to airway smooth muscle contraction in asthma. Our objective was therefore, to study the splicing regulation mechanisms of the exon5b in airway smooth muscle cells. Bioinformatics analysis was performed to identify the *cis*‐regulatory elements present in the exon5b using HSF finder 3 tool. The expression of the corresponding serine/arginine rich protein (SR) genes thus identified was evaluated by quantitative RT‐PCR (qPCR). SRSF1, SRSF6, and hnRNPA1 cis‐acting elements were identified by in silico analysis of the exon5b sequence as splicing regulator candidates. QPCR analyses showed that SRSF1 and SRSF6 are upregulated in ASM cells from asthmatic horses in exacerbation (*n* = 5) compared to controls (*n* = 5). The inhibition of the identified splicing factors by small interfering RNA allowed identifying the regulation of the SMB isoform by SRSF6. Our results implicate for the first time the upregulation of SRSF6 and SRSF1 in the asthmatic ASM cells and indicate that SRSF6 induces the exon5b inclusion. This study provides an important first step for the understanding of the triggers and pathways leading to ASM hypercontraction and identifies a possible new target for asthma.

## Introduction

Asthma is a serious public health problem with over 235 million sufferers worldwide (WHO, 2017). In Canada, 8.5% of the population (aged 12 and over) have been diagnosed as having asthma in 2010 (StatisticsCanada, 2010). Inflammation due to the asthma disease is responsible for an increase in bronchial hyperresponsiveness that causes recurrent episodes of wheezing, breathlessness, chest tightness, and cough. These episodes are marked by a variable bronchial airflow obstruction. The mechanisms leading to this airflow obstruction are poorly understood. However, smooth muscle cells have a central role in the airway obstruction. The contraction of airway smooth muscle causes the reduction of the airway's size, providing more resistance to airflow. The muscle's contraction is achieved through the interaction of two proteins; actin and myosin. Myosin is a hexametric protein composed of two myosin heavy chains and two pairs of myosin light chains and represents one of the major contractile proteins (Craig and Woodhead 2006).

Myosin heavy chain 11 (myh11) is constitutively expressed in vascular and visceral smooth muscle cells. Alternative splicing of this gene generates four different isoforms possessing different contractile properties and having differential expression levels during the cell maturation and in the various tissues (Loukianov et al. 1997; Leguillette et al. 2005). The splice site of the 3′ end region of the myh11 gene allows the production of SM1 and SM2 isoforms. SM2 isoform differs from SM1 by the inclusion of an alternative exon containing a stop codon and resulting in smaller proteins. The second alternative splice site is located in the 5′ end region of the gene in the myosin head encoding region. The inclusion of the alternative 5b exon results in the formation of the SMB isoform while its exclusion generates the SMA isoform (DiSanto et al. 1997).

In the last 20 years, the expression of SMB isoform was associated with an increased ATPase activity and speed of muscle contraction (Kelley et al. 1993; Lauzon et al. 1998; Babu et al. 2001). Indeed, a clear correlation between the SMB isoform expression and increased maximum velocity (*v*_max_) has been reported in rabbit vascular smooth muscle (DiSanto et al. 1997), in opossum esophagus (Szymanski et al. 1998), in guinea pig intestinal smooth muscle (Lofgren et al. 2002), and in canine airway (Ma and Stephens 1997). Moreover, alterations in expression level of the myh11 gene and/or the composition of myh11 mRNA isoforms have been described in numerous pathologies (Kelley et al. 1993; Karagiannis et al. 2003). In asthma, the expression level of SMB isoform was found to be 2.5 times higher in endobronchial biopsies of asthma patients compared with controls (Leguillette et al. 2009). A recent study performed Boivin et al., has shown a significantly increased expression of the SMB isoform at all levels of the bronchial tree of horses with heaves in clinical exacerbation when compared to control horses (Boivin et al. 2014). Interestingly, the expression of this fast contracting isoform, normalized when asthmatic horses are in remission of the disease.

Pre‐mRNA splicing is controlled by the spliceosome complex and is regulated by proteins that bind to intronic or exonic enhancer and silencer elements. Therefore, we hypothesized that the exonic activators and repressors could play an important role in SMB expression in the airway smooth muscle of asthmatic horses. In order to decipher the regulation of the expression levels of myh11 isoforms, the exonic enhancer elements present into the exon 5b were identified and the potential involvement of their biding proteins in myh11 SMB expression was investigated. This is a major step not only for the understanding of the triggers and pathways leading to airway smooth muscle contraction, but also for the development of new therapeutic methods in many diseases including recurrent airway obstruction and asthma.

## Methods

### Bioinformatics analysis

The Human Splicing Finder tool (http://www.umd.be/HSF3/index.html) was used to identify the auxiliary *cis*‐splicing sequences into the exon 5b of myh11. The DNA sequence used for this analysis was obtained in NCBI GenBank (*Equus caballus* genome NC009156.3: 31021290 to 31139159). The nucleotide sequence used for the analysis contains the exon 5b and five intronic bases in each the 5′ and 3′end of the exon.

### Study design

To quantify the mRNA expression of SRSF1 and SRSF6, 10 adult horses (308–800 kg, 15–28 years of age) were studied. Horses with asthma (*n* = 5) had a history of episodes of clinical exacerbation, abnormal lung function (pulmonary resistance (*R*_L_) >1 cm H_2_O/L/sec), and increased neutrophils in bronchoalveolar lavage (BAL) fluid (≥25%) upon antigen exposure consisting of stabling and hay feeding. Aged‐matched control horses (*n* = 5) had no history of respiratory diseases, and a normal lung function and BAL fluid cytology when kept in the same environment. Horses were deemed otherwise healthy, based on physical examination, and complete blood count and biochemistry profile. Controls and asthmatic horses were antigen exposed for ≥3 weeks. Animals were sacrificed, and the lungs were removed. Smooth muscle from main bronchi was manually dissected on ice, snap frozen in liquid nitrogen, and then stored at −80°C. All experimental procedures were performed in accordance with the Canadian Council for Animal Care guidelines and were approved by the Animal Care Committee for the Faculty of Veterinary Medicine of the University of Montreal (Rech‐1324). For siRNA transfection essays, ASM cells were isolated from bronchial tissue obtain from the lungs of six additional healthy horses from the slaughterhouse (Vargas et al. 2016).

### ASM cell culture and cell transfection

The cells were then seeded (50,000 cells/cm³) in DMEM/F12 (3:1) medium (Thermo Fisher Scientific, Carlsbad, CA, USA) supplemented with sodium pyruvate 100 *μ*mol/L (Thermo Fisher Scientific, Carlsbad, CA, USA), adenine 2.4 mg/L (Sigma Aldrich), 10% fetal bovine serum (FBS), penicillin‐streptomycin, and fungizone and grown in a humidified 5% CO_2_ atmosphere at 37°C. The medium was changed every 48 h, and cells were passaged at a 1:3 ratio with trypsin every 7–10 days. Cells were used between the first and fourth passage. Isolated ASM cells (0.25 × 10^5^) were first transfected with 30 pmol of each siRNA using Hiperfect reagent (301702, Qiagen) according to the manufacturer's recommendation and incubated 72 h in a humidified 5% CO_2_ atmosphere at 37°C. All siRNAs were synthesized by Thermo Fisher Scientific Canada Inc. (ID siSRSF1: s12727, ID siSRSF6: s12741, Negative Control: 4464058).

### ARN extraction and reverse transcription

To quantify the mRNA of srsf1, srsf6, and hnRNPA1 in asthmatic horses, mRNA extraction was performed on 25 mg of dissected bronchial smooth muscle using RNeasy^®^ Mini Kit (Qiagen, Toronto, ON, CA) according to the manufacturer's instructions. The qRT‐PCR was performed using specific primers (Table 1), and PCR products were sequenced to ensure the specificity of amplification products. Total RNA from siRNA‐treated ASM cells was extracted with Trizol^®^ reagent (Thermo Fisher Scientific, Carlsbad, CA, USA) then 1 *μ*g of RNA was used to generate cDNA with SuperScript™ III Reverse Transcriptase (Thermo Fisher Scientific, Carlsbad, CA, USA).

**Table 1:** Primers used for quantitative PCR

Gene name	GenBank accession number	Primers	sequence (5′–3′)
SRSF1	NM_001195548.1	SRSF1‐F SRSF1‐R	GGTCGCGACGGCTATGATTA ATCCTTCAAGTCCTGCCAGC
SRSF6	XM_023626595.1	SRSF6_F SRSF6_R	GCAAGCCTCCACTGCTTTTC CAAGGTAGACAAACCCGCCT
hnRNPA1	NM_001242509.1	HnRNPA1_F HnRNPA1_R	TTTGGCGGTGGTAGTGGAAG CTGGCTCTCCTCTCCTGCTA

### Quantitative PCR

Quantitative PCR (qPCR) was performed by monitoring the increase of fluorescence of IQ SYBR^®^ Green in real‐time (Bio‐Rad, Hercules, CA, USA) with the CFX96 Touch Real‐Time PCR Detection System and CFX Manager™ Software (Bio‐Rad, Hercules, CA, USA). The gene expression was normalized using the ribosomal protein L9 (RPL9) as reference gene. Gene expression of control siRNA‐treated ASM cells was normalized to 1. The data are presented as fold changes in mean ± SD of mRNA expression.

### Statistics

A power analysis was performed using data from a previous study (Boivin et al. 2014) and showed that five horses per group should be sufficient to detect a difference in the expression of the mhy11 exon5b *cis*‐regulatory elements. Data were analyzed with one‐way ANOVA followed by turkey post hoc test and paired *t* test, using Prism software (v6.0 g, GraphPad Software, San Diego, Ca, USA). Differences were considered significant when *P* < *0.05*.

## Results

### In silico identification of cis acting element in myh11 exon 5b

To define elements within the exon 5b of the myosin heavy chain 11 that repress or enhance the inclusion of myh11 exon 5b, we adopted a computational approach to predict splicing silencers and enhancers using HSF3 tool. The my11 exon 5b fragment contained two putative binding sites for the trans‐acting enhancer factors (SRSF1 and SRSF6) and one putative site for a trans‐acting silencer factor (hnRNPA1) (Table 1), which are known as splicing activator or repressor (Expert‐Bezancon et al. 2004). Therefore, we hypothesized that these splicing factors may repress or enhance the inclusion of my11 exon 5b.

### Expression of splicing trans‐acting factors in ASM cells of asthmatic horses

**Figure 1:**
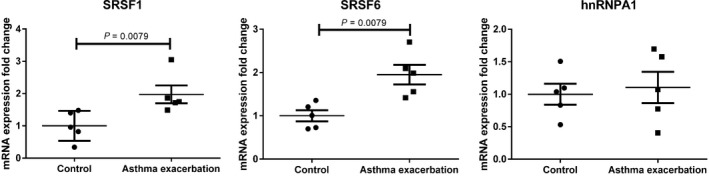
The mRNA expression of SRSF1 and SRSF6 was upregulated in asthmatic horses in exacerbation compared to controls. Quantitative Real‐time PCR were performed in triplicate using RNA from main bronchi of asthmatic horses in exacerbation (*n* = 5) or control horses (*n* = 5). The mRNA expression fold change was calculated using RPL9 as reference gene. The mRNA expression means levels observed in ASM from control horses were normalized to one. Significant differences from controls and asthmatic horses in exacerbation values were analyzed with Mann–Whitney test.

To explore the potential involvement of these three splicing factors in the regulation of the MYH11 exon 5b inclusion in asthmatic smooth muscle cells, we analyzed the mRNA expression of SRSF1, SRSF6, and hnRNPA1 in smooth muscle cells from asthmatic horses (*n* = 5) in exacerbation and in control horses (*n* = 5). Significant differences in the expression of SRSF1 and SRSF6 were observed between ASM from horses with asthma in exacerbation, when compared to controls (Fig. 1). For instance, there was a twofold increase on average in the expression of SRSF1 and SRSF6 during exacerbation of equine asthma when compared to controls (*P* = 0.008). No significant change was observed in the mRNA expression level of hnRNPA1.

### Expression of SMB myosin isoform after SRSF1 and/or SRSF6 inhibition

**Figure 2:**
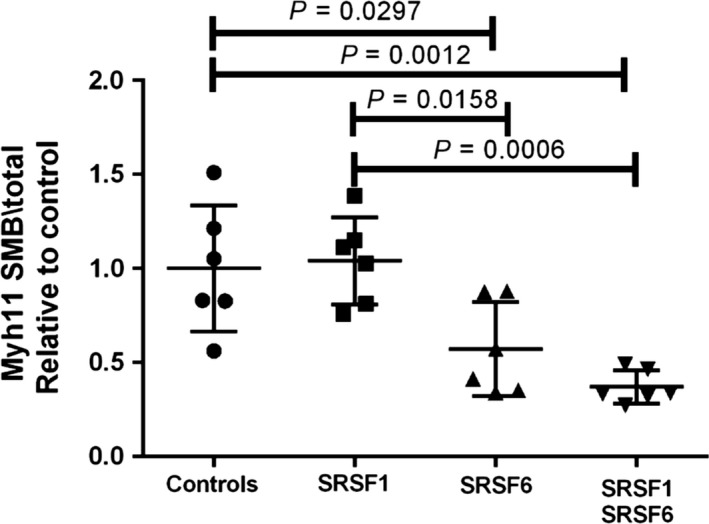
SRSF6 modulates the expression of the myh11 SMB isoform. The inhibition of SRSF6 by siRNA diminished the expression of the myh11 SMB isoform. For each sample RT‐PCR were performed in triplicate. The experiment was conducted using cDNA from six distinct ASM cell cultures of six distinct horses. Significant difference from control values were analyzed with the one‐way ANOVA followed by Tukey post hoc test.

We next investigated whether SRSF1 and SRSF6 were involved in enhancing the inclusion of exon 5b. We used siRNAs to downregulate each protein individually or both proteins simultaneously. A decrease in endogenous myh11 exon 5b inclusion was not detected when SRSF1 was depleted (Fig. 2). Interestingly, a decrease in exon 5b inclusion was detected when either SRSF6 only or SRSF1 and SRSF6 together were depleted compared to control siRNA (*P* = 0.03 and *P* = 0.001, respectively).

## Discussion

Our recent work demonstrated that myh11 SMB isoform is increased in asthmatic horses (Boivin et al. 2014). Overexpression of the SMB isoform in smooth muscle cells led to increase of the smooth muscle velocity, and could account for altered contractile properties observed in human pathology including asthma (Leguillette et al. 2005). However, the splicing mechanism of this isoform is unknown. In this study, we describe the splicing *cis*‐acting elements within the exon 5b nucleotide sequence. The biding site of the serine/arginine (SR) protein SRSF6 that enhance inclusion of exon 5b and was upregulated in asthmatic airway smooth muscle cells. To our knowledge, this study is the first to identify splicing *cis*‐acting elements involved in the regulation of myh11 exon 5b splicing.

The in silico analysis of the myh11 exon 5b sequence allowed to identify three *cis*‐acting elements corresponding to the binding sites of the splicing factors, SR protein SRSF1 (SF2/ASF), SRSF6 (SC35), and the heterogeneous nuclear ribonucleoprotein A1 (hnRNPA1) (Table 2). These splicing factors are important regulators of mRNA constitutive and alternative pre‐mRNA splicing (Long and Caceres 2009). HnRNPA1 is known to bind to exonic silencer element and repress the splicing of alternative exon and had antagonist activity against the splicing factor enhancers SR proteins (Del Gatto‐Konczak et al. 1999; Zhu et al. 2001; Caputi and Zahler 2002). The differential expressions of SRSF1, SRSF6, and hnRNPA1 in different cell types affect the alternative splicing of many pre‐mRNA and regulation of these splicing factors mRNA expression is known as one of the mechanisms involved in exon inclusion or skipping (Mayeda et al. 1993; Caceres et al. 1994; Hanamura et al. 1998; Rooke et al. 2003).

**Table 2:** Identification of *cis*‐acting splicing factor in myh11 exon 5b using the Human Splicing Finder tool

Splicing factor	Position	Pattern	Score	Threshold value
SRSF1	+6	CTGCCTA	78	70.5
SRSF6	+12	TGCTCA	79	74
hnRNPA1	+2	AAGGCC	73	65.5

The analysis of the mRNA expression showed the upregulation of SRSF1 and SRSF6 in the airway smooth muscle of asthmatic horses in exacerbation compared to control (Fig. 1). The overexpression of these SR proteins could regulate the exon splicing in asthmatic horses, including myh11 exon 5b that contain their *cis*‐acting elements. In order to validate this result at protein levels, commercial available antibodies were test in preliminary experiments. Unfortunately, they did not cross react with SRSF1 and SRSF6 from horses. However, this result leads us to hypothesize that SRSF1 and SRSF6 are involved in myh11 exon 5b splicing in airway smooth muscle. To verify this hypothesis, we repressed the expression of SRSF1 and SRSF6 mRNA using siRNA and analyzed the mRNA expression of myh11 SMB isoform. Our results show that the expression of myh11 SMB isoform is modulated by SRSF6 (Fig. 2). However, myh11 SMB expression was not modulated by SRSF1 and hnRNPA1. The upregulation of SR proteins was described in many pathologies including lung and colon cancer (Cohen‐Eliav et al. 2013). These *trans*‐regulatory proteins are not specific to contractile proteins as they are also involved in the splicing of other genes including different growing factors (Fan et al. 2009; Xie et al. 2017). Interestingly, in vascular smooth muscle cells, SRSF1 is overexpressed, while SRSF1 knockdown suppresses the proliferation and migration of cultured human aortic and coronary arterial smooth muscle cells (Xie et al. 2017). Thereby, these regulatory proteins could play a central role in the dysregulation of proliferation and contractility observed in the asthmatic airway smooth muscle cells. Moreover, our results could have a large impact, because the myh11 SMB isoform is also involved in many human pathologies including intestinal and urinary obstruction or hypertension (Wetzel et al. 1998; Karagiannis et al. 2003).

Taken together, these results suggest an important role of splicing factors in airway smooth muscle remodeling in asthma. A better understanding of the role of these splicing factors in asthma may contribute to the development of novel therapy to reverse bronchial smooth muscle remodeling.

## Conflict of Interest

The authors declare no competing financial interests.
